# Novel Fluorescent Nanobiosensors for Rapid and Sensitive Detection of Organophosphorus Pesticide Residues in *Angelica sinensis*: A Performance Evaluation Against LC-MS

**DOI:** 10.3390/bios16060311

**Published:** 2026-06-01

**Authors:** Xiqiong Mu, Yaqin Dong, Ling Jin, Tiantian Zhu

**Affiliations:** 1 College of Pharmacy, Gansu University of Chinese Medicine, Lanzhou 730000, China; m15214046636@163.com (X.M.); 18893073722@163.com (Y.D.); 2 Ministry of Education and Provincial Co-Construction Collaborative Innovation Center for Northwest Chinese and Tibetan Medicine, Lanzhou 730000, China; 3 Gansu Provincial Engineering Research Center for Evaluation, Protection and Utilization of Rare Traditional Chinese Medicine Resources, Lanzhou 730000, China; 4 Long Medicinal Materials Industry Innovation Research Institute, Lanzhou 730000, China

**Keywords:** *Angelica sinensis*, organophosphorus pesticide residues, fluorescent nanosensor, liquid chromatography–mass spectrometry

## Abstract

This study compares the performance of novel fluorescent nanobiosensors and liquid chromatography–mass spectrometry (LC-MS) for detecting organophosphorus pesticide residues in *Angelica sinensis*. Key parameters such as recovery rate and relative standard deviation (RSD) were evaluated to identify a preferable detection method. The results show that the fluorescent nanobiosensor offers superior sensitivity, whereas LC-MS excels in accuracy and quantitative analysis. For phoxim, the GQDs@GSH sensor achieved an LOD of 0.075 μmol·L^−1^, with recoveries of 97.65–100.62% (RSD < 3.35%). For glyphosate, the PDOA/Cu^2+^ sensor achieved an LOD of 1.8 nmol·L^−1^, and the AgNCs sensor achieved an LOD of 21 nmol·L^−1^, with recoveries of 91.30–105.34% (RSD < 3.35%). The originality of this work is threefold and does not claim de novo synthesis of unknown materials: (i) it is the first validation of three existing fluorescent nanosensors (GQDs@GSH, PDOA/Cu^2+^, and AgNCs) specifically for organophosphorus pesticide detection in the complex medicinal plant matrix *Angelica sinensis*; (ii) it provides the first systematic comparison of these nanosensors against the pharmacopeia-standard LC-MS method in this matrix, delineating their complementary advantages; (iii) it is the first assessment of their phytotoxicity in *Angelica sinensis* seedlings, demonstrating biocompatibility and potential for in planta imaging.

## 1. Introduction

*Angelica sinensis* (Oliv.) Diels, a perennial herb in the Apiaceae family, is derived from the dried root of the plant and is widely utilized in traditional Chinese medicine and culinary applications [1,2]. It is also a significant export product among Chinese herbal commodities. As a medicinal and edible herb of great importance, the quality and safety of *Angelica sinensis* have garnered considerable attention. The expansion of cultivation areas, establishment of large-scale production zones, and extended cultivation periods have led to increasing pest and disease pressures. Managing these issues poses significant challenges, including diverse application methods, excessive pesticide use, soil barriers, control difficulties, and inconsistent efficacy.

Organophosphorus pesticides (OPPs) are commonly employed in *Angelica sinensis* cultivation due to their high efficacy, broad spectrum, affordability, and they contribute to improved quality and yield [3]. However, the improper application of OPPs leads to residue accumulation in the soil and subsequent human exposure through skin contact, inhalation, or ingestion, causing acetylcholinesterase poisoning [4]. This not only endangers human health but also affects the production and marketability of *Angelica sinensis*. Consequently, developing an efficient, simple, accurate, sensitive, and stable method for detecting pesticide residues is critical for preventing and mitigating pesticide pollution.

The liquid chromatography–mass spectrometry (LC-MS) method, as prescribed in the 2020 edition of the Chinese Pharmacopeia, is a widely used technique for organophosphorus pesticide detection and offers distinct advantages [5]. LC-MS enables qualitative and quantitative analyses of organic compounds with high molecular weights, low volatility, and thermal instability [6]. Compared to standalone liquid chromatography or mass spectrometry, LC-MS significantly enhances sensitivity, selectivity, and accuracy. However, the method requires costly instruments, large operational spaces, strict maintenance protocols, and skilled personnel. Furthermore, sample preparation processes such as separation and purification are complex, limiting their application for the rapid on-site detection of pesticide residues in *Angelica sinensis* [7].

In recent years, nanoscale sensors have emerged as a promising technology for detecting organophosphorus pesticide residues in *Angelica sinensis*. These sensors are characterized by compact size, rapid response, high sensitivity, excellent integration, and the ability to provide clear three-dimensional cellular imaging [8,9,10]. Recent advances have further expanded their applications, including thermo-fluorescent bactericidal quantum dots for smart textiles [11] and wearable electrochemical biosensors for disease diagnosis [12]. Such properties make them highly effective for on-site detection of trace and ultratrace pesticide residues in traditional Chinese herbal medicines. Compared to traditional methods outlined in the Chinese Pharmacopeia, nanoscale sensors offer advantages such as lower cost, simplified pretreatment, shorter detection times, and lower detection limits.

Various nanoscale sensing technologies have been developed for pesticide residue monitoring. Li et al. [13] established a sensitive fluorescence detection method for dimethoate based on enzyme inhibition and copper-triggered oxidation of o-phenylenediamine (OPD). Zhang et al. [14] synthesized a near-infrared fluorescent probe, HP-LZB, capable of specifically detecting butyrylcholinesterase (BChE) in live cells, thereby enabling the study of interactions between organophosphate compounds (OPs) and endogenous BChE. Similarly, Wang et al. [15] developed a nitrogen-doped carbon quantum dot (N-CQDs)-based sensor using the acetylcholinesterase inhibition mechanism for rapid detection of OPP residues. While these sensors exhibit excellent detection performance, most have not been applied specifically for detecting OPP residues in *Angelica sinensis*.

Our research team previously synthesized fluorescent sensors with signal amplification capabilities, including glutathione-modified GQDs@GSH, AgNCs, and PDOA nanoparticles [16,17,18]. We investigated their mechanisms of action, structure–performance relationships, and regulatory factors to design fluorescent nanobiological sensors for detecting OPP residues in *Angelica sinensis*. These sensors enabled real-time dynamic imaging analysis of OPP residues with high sensitivity. Despite their excellent detection performance, comparative studies between fluorescent nanosensors and LC-MS for detecting OPP residues in *Angelica sinensis* remain lacking, limiting the persuasiveness of current findings.

In this study, we focused on phoxim, a commonly used organophosphorus pesticide in *Angelica sinensis* cultivation, and glyphosate, a widely applied herbicide. We compared the performance of three types of fluorescent nanosensors with LC-MS. This investigation not only provides a feasible solution for rapid on-site detection of pesticide residues in *Angelica sinensis* but also serves as a reference for analyzing other pesticide residues. This study represents the first application of GQDs@GSH, PDOA, and AgNCs for the specific detection of phoxim and glyphosate residues in *Angelica sinensis*. It is also the first systematic comparison of these nanosensors with the standard LC-MS method in this complex herbal matrix, and the first assessment of their phytotoxicity in *Angelica sinensis* seedlings.

## 2. Materials and Methods

### 2.1. Reagents

3,4-Dihydroxyphenylalanine was purchased from Sigma-Aldrich Reagent Company (Shanghai, China). N-(2-Hydroxyethyl)piperazine-N′-ethanesulfonic acid (HEPES) was obtained from Aladdin Chemical Company (Shanghai, China). Poly (methacrylic acid, sodium salt) (PMAA, average Mw = 6000) was supplied by Sigma-Aldrich Reagent Company (Shanghai, China). Glyphosate was purchased from J&K Scientific (Shanghai, China). Phoxim (purity > 95%) was purchased from Shanghai McLean Biochemical Technology Co., Ltd. (Shanghai, China). Other chemical reagents were of analytical grade and purchased from Tianjin Baishi Chemical Co., Ltd. (Tianjin, China).

### 2.2. Angelica sinensis

The test plants used in the study were 1-year-old *Angelica sinensis* (Oliv.) Diels seedlings, provided by the Minxian County Chinese Medicine Production Technology Guidance Station. These seedlings were identified as members of the Apiaceae family by Professor Wang Yinquan from Gansu University of Traditional Chinese Medicine.

The growth season of *Angelica sinensis* spans from April to October. A field control trial was conducted during this period at the green standardized planting base for traditional Chinese medicine in Habancha Village, YuZhong County, Lanzhou City, Gansu Province (35°46′ N, 103°59′ E). This site is approximately 50 km southeast of Lanzhou and 15 km southwest of YuZhong County, located within a high-altitude, semi-shaded mountainous region. The trial was carried out from August to September 2023. The area, situated at an elevation of 2663 m, experiences an annual average temperature of 3.2 °C, a frost-free period of about 184 days, and an annual sunshine duration of 2214.9 h. The average annual precipitation is 550 mm, most of which falls between May and August.

Three treatments were included: phoxim (PHO), glyphosate (GLY), and a control (CK), arranged in a randomized complete block design with three replicates. Each plot measured 2 m × 3 m, and plots were separated by 0.5 m buffer zones to prevent cross-contamination. Root irrigation was applied every 7 days starting 20 August 2023 for a total of four applications. The dosages of PHO and GLY were selected based on standard agricultural practice and adjusted for the specific needs of this study.

The snake sampling method was employed to collect *Angelica sinensis* plants. The entire plant was carefully excavated from the soil, and loose soil adhering to the root system was removed by shaking. Subsequently, tightly attached soil was brushed off, and parts outside the roots were trimmed away, leaving sufficient material as a root sample. The root samples underwent identical processing, with each divided into two portions and sealed in bags for rapid transportation to the laboratory. One portion was used to measure plant morphology data, while the other was naturally air-dried and preliminarily ground to determine organophosphorus pesticide residues in the roots.

### 2.3. Detection and Evaluation of Nanoscale Sensors

#### 2.3.1. Synthesis of Fluorescent Sensors

The three nanosensors were synthesized following our previously reported protocols [16,17,18] with minor modifications. Detailed characterization data are provided below and in the Supplementary Materials.

GQDs@GSH: Glutathione-modified graphene quantum dots (GQDs) were synthesized via a one-pot hydrothermal method using citric acid as a carbon source and glutathione as a surface passivation agent (see Supplementary Materials, Figure S1 for schematic). The obtained GQDs@GSH exhibit bright blue fluorescence (excitation 360 nm, emission 450 nm) with a quantum yield of 28.6%. Fluorescence quenching by Fe^3+^ occurs through a photoinduced electron transfer (PET) mechanism, confirmed by time-resolved lifetime analysis. Subsequent addition of phytic acid (PA) restores fluorescence by reducing Fe^3+^ to Fe^2+^ and forming a stable PA–Fe^2+^ complex. In the presence of acetylcholinesterase (AChE) and choline oxidase (ChOx), the hydrolysis of acetylcholine (ACh) generates H_2_O_2_, which re-oxidizes Fe^2+^ to Fe^3+^, leading to fluorescence re-quenching. Phoxim inhibits AChE, preventing H_2_O_2_ production and thereby maintaining the “on” state. This cascade enables sensitive phoxim detection. Initial calibration curves were prepared using solvent standards in buffer solutions. For real *Angelica sinensis* extract samples, the standard addition method was employed to compensate for matrix effects. This cascade “off-on-off” mechanism enables sensitive detection of phoxim.

PDOA/Cu^2+^: Dopamine-modified fluorescent probes were developed for glyphosate detection. The fluorescence of the probe, quenched by copper ions (Cu^2+^), was restored upon the interaction of glyphosate with Cu^2+^.

AgNCs: Silver nanoclusters (AgNCs) were utilized for glyphosate detection based on glyphosate-induced aggregation, which resulted in significant fluorescence quenching.

#### 2.3.2. Plant Toxicity Evaluation

The phytotoxicity of the GQDs@GSH, AgNCs, and PDOA fluorescent probes was evaluated using *Angelica sinensis* seedlings. Seeds of similar size were divided into a control group and three experimental groups. The experimental groups were treated with 5 mg·L^−1^ GQDs@GSH, 1.2 mg·mL^−1^ AgNCs, and 3.4 mg·mL^−1^ PDOA solutions, respectively, while the control group was cultured in distilled water without any additives. After 14 days of culture, healthy seedlings were washed with triple-distilled water. Growth parameters, including plant height (measured from the ground to the plant apex) and root width (the maximum root diameter), were measured using a digital vernier caliper with an accuracy of 0.01 mm. Fluorescence imaging of the roots was performed using a fluorescence microscope (DMI6000B, Leica, Wetzlar, Germany) to observe any structural changes. The instrument settings were as follows: 20× objective lens and a green light filter (serial No. 4). Images were captured using ECHO PRO software. The results from the experimental groups were compared with those of the control group to assess the potential toxic effects of each fluorescent probe.

### 2.4. LC-MS Detection of PHO Residues

#### 2.4.1. Standard Curve Preparation

PHO standard solutions were prepared at a stock concentration of 100 mg/L in acetonitrile and further diluted to working standard solutions at concentrations of 0.1, 0.5, 1, 5, and 10 mg/L. The calibration curve was constructed by plotting peak area against concentration, yielding a correlation coefficient (R^2^) ≥ 0.99, which ensured accurate quantification.

#### 2.4.2. Sample Preparation

Root samples of Angelica sinensis were freeze-dried and ground to a fine powder. Pesticide residues were extracted using the QuEChERS (Quick, Easy, Cheap, Effective, Rugged, and Safe) method. Briefly [17]:

Extraction: 1.0 g of powdered root sample was homogenized in 10 mL of acetonitrile, followed by the addition of 4 g of anhydrous MgSO_4_ and 1 g of NaCl. The mixture was vortexed for 1 min and centrifuged at 4000 rpm for 5 min.

Purification: The supernatant was purified using dispersive solid-phase extraction (SPE) with 150 mg of octadecylsilane (C_18_) sorbent. After mixing and centrifugation, the purified extract was evaporated to dryness under nitrogen and redissolved in 1 mL of acetonitrile for analysis.

#### 2.4.3. Chromatographic Conditions

LC-MS analysis was performed on a ThermoFisher UltiMate 3000 HPLC system (Thermo Fisher Scientific, Waltham, MA, USA). Phoxim detection was carried out using a Thermo Scientific™ Accucore™ aQ C_18_ polar-capped LC column (10 cm × 2.1 mm, 2.6 µm). The mobile phase consisted of 0.1% formic acid containing 5 mmol·L^−1^ ammonium formate (phase A) and water (phase B). Gradient elution was performed as follows: 0 min (40% A), 5 min (90% A), 10 min (40% A), and 15 min (40% A). The flow rate was set at 0.2 mL·min^−1^, and the column temperature was maintained at 40 °C.

#### 2.4.4. Mass Spectrometry Conditions

The mass spectrometry analysis was performed using a triple quadrupole tandem mass spectrometer (Agilent Technologies, Inc., Shanghai, China) equipped with an ESI ion source in positive ion scanning mode. The spray voltage was set at 3200 V, with a capillary temperature of 300 °C. The sheath gas flow rate was 40 arbitrary units (Arb), and the auxiliary gas flow rate was 8 Arb. The maximum injection current was maintained at 100 µA, and the probe heater temperature was set to 300 °C.

#### 2.4.5. Field Sample Detection

Field-grown root samples were prepared and analyzed under identical conditions to ensure data consistency.

### 2.5. LC-MS Detection of GLY Residues

#### 2.5.1. Standard Curve and Calibration for GLY

GLY standard stock solutions (100 mg/L) were diluted to concentrations of 0.1, 0.25, 0.5, 1, and 5 mg/L. Calibration curves were constructed using peak area against concentration, achieving R^2^ ≥ 0.99.

#### 2.5.2. Glyphosate Sample Preparation

Glyphosate in Angelica sinensis root samples was extracted and derivatized for LC-MS analysis [18]. Extraction: 1.0 g of root powder was mixed with 10 mL of 0.1 M HCl, vortexed for 1 min, and sonicated for 15 min. The mixture was centrifuged at 4000 rpm for 10 min, and the supernatant was collected.

Derivatization: A 1 mL aliquot of the extract was reacted with 1 mL of 10 mM 9-fluorenylmethyl chloroformate in acetonitrile at room temperature for 30 min, followed by the addition of 2 mL of 0.1 M phosphate buffer (pH 7.0). The derivatized solution was filtered through a 0.22 μm membrane for analysis.

#### 2.5.3. Chromatographic Parameters

The chromatographic analysis was conducted using a ZORBAX SB-AQ Agilent column (Agilent Technologies, Santa Clara, CA, USA; 15 cm length, 4.6 mm inner diameter, 5.0 µm particle size), packed with octylsilane-bonded silica gel. Methanol was used as solvent A, and 0.1% formic acid in water was used as solvent B, with a solvent ratio of A:B = 1:99. Gradient elution was performed with phase A set to 40–90–40–40. The flow rate was maintained at 0.3 mL/min, and the column temperature was kept at 15. The optimized MRM parameters for phoxim and glyphosate are presented in Table 1.

#### 2.5.4. MS Parameters

Mass spectrometry detection was carried out using a triple quadrupole tandem mass spectrometer equipped with an ESI ion source. The spray voltage was set at 3200 volts, with a capillary temperature of 300 °C. The sheath gas flow rate was 40 Arb, and the auxiliary gas flow rate was 8 Arb. The maximum injection current was 100 µA, and the probe heater temperature was maintained at 300 °C.

### 2.6. Data Analysis

Data from three replicates were analyzed using one-way ANOVA (IBM SPSS Statistics 26) and visualized with Origin 2018. A significance level of *p* < 0.05 was applied. For field-grown samples, ‘Residue/Applied (%)’ in Table 2 is defined as measured group comparisons; *p*-values < 0.05 were considered statistically significant residue concentration divided by applied pesticide dose × 100%. ANOVA and paired *t*-test were used for (see Section 3.2).

For field-grown samples, “Residue/Applied (%)” is defined as the measured pesticide concentration in roots divided by the total applied dose, expressed as a percentage, indicating the detectable residue proportion at harvest—distinct from analytical spike recovery. The limit of detection (LOD) and limit of quantification (LOQ) for both the fluorescent nanosensors and LC-MS method were calculated as 3σ/S and 10σ/S, respectively, where σ is the standard deviation of the response (derived from blank signals or residual standard deviation of the regression line), and S is the slope of the calibration curve obtained using matrix-matched standards in *Angelica sinensis* root extract.

## 3. Results and Discussion

### 3.1. Evaluation of Plant Toxicity of GQDs@GSH, AgNCs, and PDOA Fluorescent Probes

The fluorescent probes GQDs@GSH, AgNCs, and PDOA demonstrate a notable impact on the growth of *Angelica sinensis*, with exceptional optical properties that make them ideal for high-resolution biological imaging. In this study, a control group was cultivated using pure water, while three experimental groups were treated with solutions containing the fluorescent probes GQDs@GSH, PDOA, or AgNCs.

**Figure 1:**
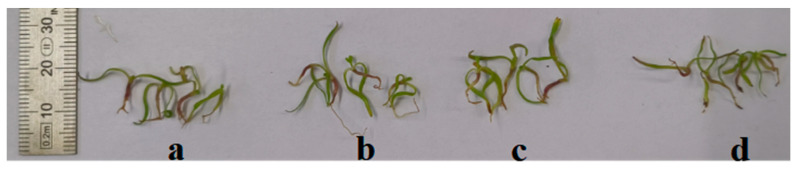
(a) Effects of different fluorescent probes on the seed growth of *Angelica sinensis* after 14 days of culture. (**a**) Control group; (**b**) GQDs@GSH; (**c**) AgNCs; (**d**) PDOA.

As shown in Figure 1, after 14 days of cultivation, seedlings in all groups successfully germinated and developed hypocotyls. Compared with the control group, the experimental groups treated with the fluorescent probes exhibited increased plant height and root width, suggesting that these probes promote the growth of *A. sinensis* seedlings. These results indicate that GQDs@GSH, PDOA, and AgNCs do not exert any detrimental phytotoxic effects on seedling development.

**Figure 2:**
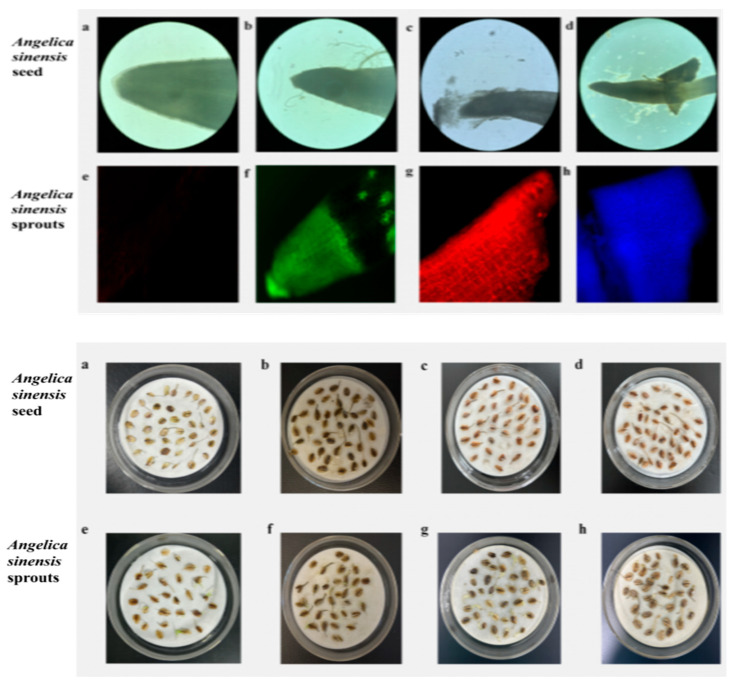
Growth curves of unsprouted and sprouted *Angelica sinensis* seeds cultured with different fluorescent probes ((**a**,**e**): control group; (**b**,**f**): GQDs@GSH; (**c**,**g**): AgNCs; (**d**,**h**): PDOA). Fluorescence images of live imaging of *Angelica sinensis* seeds treated with different fluorescent probes ((**a**,**e**): control group; (**b**,**f**): GQDs@GSH; (**c**,**g**): AgNCs; (**d**,**h**): PDOA).

Fluorescence microscopy imaging (Figure 2) revealed strong fluorescence signals corresponding to the characteristic emission wavelengths of each probe: Blue fluorescence in the blue channel for GQDs@GSH, red fluorescence in the red channel for PDOA, and green fluorescence in the green channel for AgNCs.

These findings confirm that the fluorescent probes can effectively penetrate the root tip tissues of *Angelica sinensis*. A closer examination of the root tips showed uniform staining of root tip cells, with higher fluorescence intensity concentrated in the root tip regions. This suggests that the probes are internalized via endocytosis at the root tips and subsequently transported throughout the root tissues, demonstrating selective distribution within the plant.

Importantly, fluorescence imaging revealed that the root tip cells maintained their structural integrity, with no observable damage despite substantial uptake of the fluorescent probes. This indicates that the probes exhibit high biocompatibility, allowing *Angelica sinensis* cells to remain viable during staining.

In conclusion, these results demonstrate that GQDs@GSH, PDOA, and AgNCs are non-phytotoxic and can serve as efficient fluorescent markers for bioimaging applications in *Angelica sinensis*. Their selective uptake and strong fluorescence properties make them promising tools for plant imaging and related biological studies.

### 3.2. Detection of Phoxim Residues in Angelica sinensis

#### 3.2.1. LC-MS Calibration Curve

**Figure 3:**
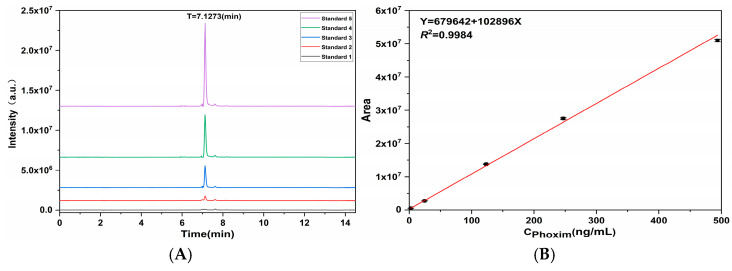
Overview of the detection and quantification of phoxim using LC-MS. (**A**) Total ion chromatogram of phoxim at different concentrations; (**B**) standard curve of phoxim, plotting the peak area (y) of the quantitative ion against its concentration (x, μmol·L^−1^).

Figure 3A shows that the retention time for various concentrations of phoxim standards in total ion chromatography is 7.1273 min. The standard curve of the quantitative ion peak area of phoxim (y) versus its concentration (x, μmol·L^−1^) is illustrated in Figure 3B. Using the least squares method, the regression equation was determined as y = 679,642 + 102,896x. The response values for synthetic phoxim and its metabolites within the Anglica sinensis matrix displayed a strong linear correlation with concentrations, with R^2^ > 0.99, confirming the method’s reliability for phoxim quantification across the tested concentration range.

#### 3.2.2. LC-MS Spiking Detection Method

**Figure 4:**
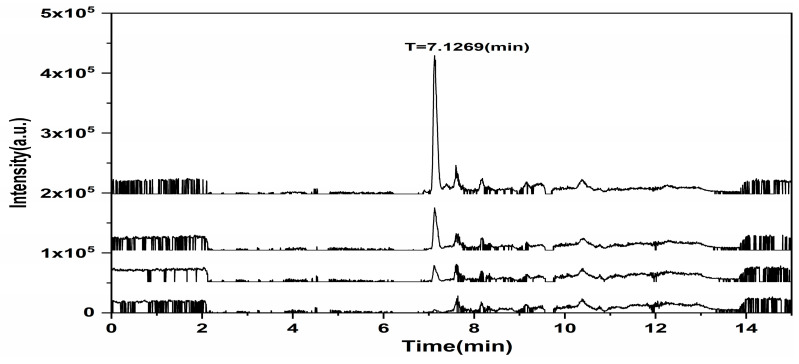
Total ion flow chromatogram of phoxim in the *Angelica sinensis* extract analyzed using the standard addition method.

As depicted in Figure 4, the retention time of phoxim in the *Angelica sinensis* extract was 7.1267 min, differing by only 0.0006 min from the standard solutions. This close match confirms that phoxim detection based on retention time within ±0.2 min is valid. Using the standard addition method, phoxim recovery was tested at three levels (low, medium, and high) in a blank matrix of *Angelica sinensis* extract free of pesticide residues.

For the GQDs@GSH sensor system, recovery rates ranged from 97.65% to 100.62%, with relative standard deviations (RSD) between 0.49% and 3.35%. By contrast, LC-MS quantification yielded recovery rates of 11.12% to 25.46%, with RSDs of 0.25% to 3.78%. A strong matrix effect (ME = −72%) was observed for phoxim due to ion suppression caused by co-extracted interferents in *Angelica sinensis* root extract, consistent with previous reports on complex herbal matrices. In contrast, the GQDs@GSH nanosensor was unaffected by matrix effects, achieving excellent recoveries (97–100%) under identical spiked conditions. This phenomenon does not invalidate LC-MS as a gold standard. Rather, it delineates its boundary condition: LC-MS requires extensive sample cleanup to mitigate matrix effects. In contrast, the GQDs@GSH nanosensor, relying on fluorescence signal transduction rather than ionization, is inherently immune to ion suppression. This contrast defines the complementary value of the two platforms. LOD and LOQ were calculated as 3σ/S and 10σ/S, respectively, based on matrix-matched calibration curves in accordance with ICH Q2 (R1) guidelines. The LOQ was experimentally validated at the lowest spiked concentration (0.146 μmol·L^−1^), where recoveries ranged from 91.30% to 105.34% with RSD < 3.35%, confirming reliable quantification. Notably, at a phoxim concentration of 0.0414 μmol·L^−1^, the GQDs@GSH biosensor successfully detected residues, while the LC-MS method failed. This demonstrates that the GQDs@GSH biosensor system has a lower detection limit and is more suitable for identifying low concentrations of phoxim in *Angelica sinensis*. The GQDs@GSH sensor also has low detection limits and recovery rates compared to other methods [19]. A paired *t*-test confirmed that the difference between GQDs@GSH and LC-MS recoveries was statistically significant at low concentrations (*p* < 0.01), while no significant difference was observed at medium and high concentrations (*p* > 0.05).

#### 3.2.3. Determination of Phoxim Pesticide Residues in Field-Grown *Anglica sinensis* Samples

**Figure 5:**
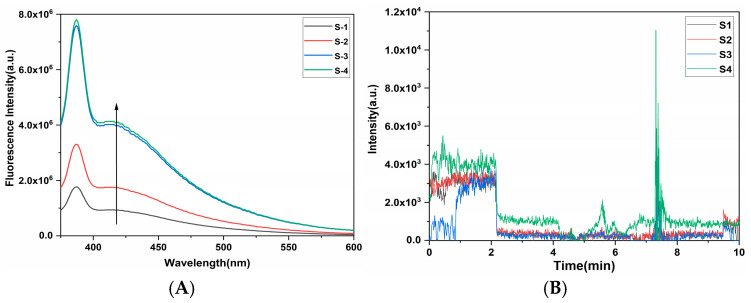
(**A**) Fluorescence spectrum of phoxim in *Angelica sinensis* using the GQDs@GSH sensor system; (**B**) total ion chromatogram of Phoxim in *Angelica sinensis* detected by LC-MS. Note: Arrow: intensity variation across samples.

The practicality of the GQDs@GSH sensor system for real-world applications was assessed by comparing its performance with LC-MS in detecting phoxim residues in field-grown *Angelica sinensis* samples. Figure 5 shows the results of quantitative screening conducted using the GQDs@GSH system (Figure 5A) and LC-MS (Figure 5B).

Table 3 presents the recovery rates for phoxim residues in four batches of samples. The GQDs@GSH sensor system detected concentrations ranging from 0.0384 to 1.383 μmol·L^−1^, with recovery rates between 0.12% and 1.39%. The LC-MS method also detected phoxim in the last three batches, with recovery rates from 0.66% to 1.39%. However, the GQDs@GSH system uniquely detected phoxim in Sample S1 at a concentration of 0.0384 μmol·L^−1^, which was below the detection limit of LC-MS.

These results reaffirm the lower detection limit and higher sensitivity of the GQDs@GSH system, making it particularly effective for detecting low phoxim concentrations in *Angelica sinensis*. In this study, the recovery rate is defined as the residual rate of pesticides in plants, representing a key parameter in research on the environmental behavior of pesticides. Specifically, this metric reflects the proportion of the applied pesticide that remains detectable in root tissues at harvest. The observed values (0.12–1.39% for phoxim) are consistent with typical field dissipation rates for organophosphorus pesticides and serve as a measure of plant uptake and accumulation. The relatively low residue levels detected in field samples may be attributed to several possible factors, such as dilution by rainfall during the growing season or metabolic degradation within the plant. However, the absence of direct environmental measurements or metabolite analyses precludes confirmation of these hypotheses. The findings suggest that the GQDs@GSH/PA/(ACh + AChE + ChOx) fluorescent nanobiosensor is a robust tool for residue detection, particularly in low-concentration scenarios, offering a reliable alternative to conventional LC-MS techniques.

#### 3.2.4. Sensing Mechanism of GQDs@GSH for Phoxim

The fluorescence quenching of GQDs@GSH by Fe^3+^ is attributed to a photoinduced electron transfer (PET) process, as confirmed by time-resolved fluorescence lifetime analysis (τ decreased from 2.08 ns to 1.19 ns). Phytic acid (PA) restores fluorescence by reducing Fe^3+^ to Fe^2+^ and forming a PA/Fe^2+^ complex (binding constant K = 1.2 × 10^4^ M^−1^). AChE/ChOx-catalyzed ACh hydrolysis produces H_2_O_2_, which re-oxidizes Fe^2+^ to Fe^3+^, quenching fluorescence again. Phoxim inhibits AChE (IC_50_ = 18.5 nM under assay conditions), preventing H_2_O_2_ production and restoring fluorescence. This “off-on-off” mechanism allows signal amplification with a detection limit of 0.075 μmol·L^−1^ in the *Angelica sinensis* matrix. For comparison, derivatization-LC-MS methods for organophosphorus pesticides typically achieve higher selectivity but require longer sample preparation [20] Similar fluorescence switchable behavior based on Fe^3+^ quenching and subsequent recovery has been reported in other GQD systems [21].

### 3.3. Determination of Glyphosate Residues in Anglica sinensis

#### 3.3.1. LC-MS Standard Curve Plotting

**Figure 6:**
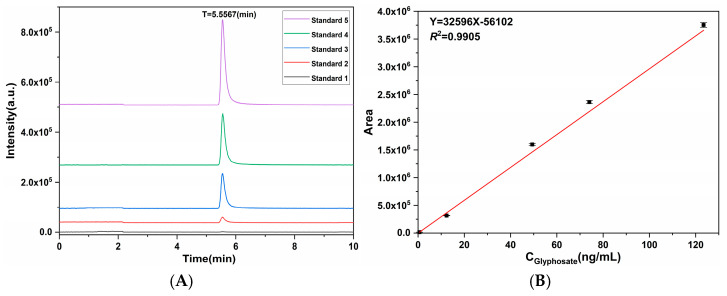
(**A**) Total ion chromatogram of glyphosate at different concentrations; (**B**) glyphosate standard curve. Note: The red solid line shows the best linear fit of the calibration curve, and the symbols (■) represent the mean peak areas (n = 3) at each glyphosate concentration.

As shown in Figure 6A, the retention time for glyphosate standard solutions at various concentrations in the total ion chromatogram was 5.5567 min. The standard curve, plotting the peak area (y) of the glyphosate quantitative ion against its concentration (x, μmol·L^−1^), is presented in Figure 6B. The regression equation, derived using the least squares method, was y = 32,596x − 56,102. The response values for glyphosate and its metabolites in the *Angelica sinensis* matrix showed a strong linear correlation with concentration, with *R*^2^ > 0.99 being within the specified concentration range, indicating a highly reliable and accurate method for glyphosate quantification.

#### 3.3.2. Marking Detection Method

**Figure 7:**
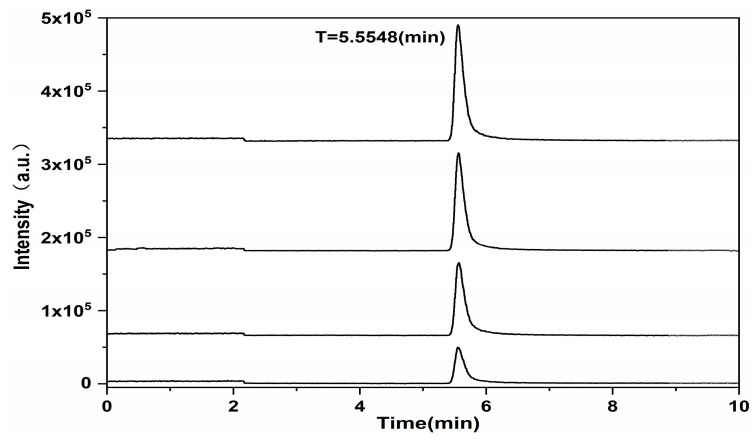
Total ion chromatogram of glyphosate in *Angelica sinensis* extract measured using the derivatization method.

To evaluate the accuracy and precision of the developed sensing methods (AgNCs, PDOA) for detecting glyphosate in real samples, we applied the standard addition method to *Angelica sinensis* extract samples. Glyphosate was spiked into the samples at concentrations of 0.1460, 0.2190, 0.2920, and 0.3650 µmol·L^−1^, followed by analysis. As shown in Figure 7, the retention time of glyphosate in the *Angelica sinensis* extract was 5.5548 min, differing by only 0.0019 min from the retention time of the corresponding standard solutions at various concentrations of glyphosate (Figure 6). This small deviation satisfies the criterion that the difference in retention time between the target analyte in the sample and its corresponding standard solution should be within ±0.2 min for a positive identification.

As shown in Table 4, the recovery rates of *Angelica sinensis* samples spiked with standards and analyzed using the developed sensing system (PDOA, AgNCs) ranged from 99.27% to 105.34% for PDOA and from 91.30% to 101.29% for AgNCs, with all relative standard deviations (RSDs) remaining below 3.35%. To further validate the accuracy of the method, the glyphosate content in the spiked *Angelica sinensis* samples was quantified using LC-MS. A paired *t*-test revealed a significant difference (*p* < 0.05) between the sensor system (PDOA, AgNCs) and LC-MS detection results only at the low concentration of glyphosate (0.146 µmol·L^−1^) in *Angelica sinensis* samples. LOD and LOQ for glyphosate detection by PDOA/Cu^2+^ and AgNCs sensors were calculated as 3σ/s and 10σ/s, respectively, using matrix-matched calibration curves in *Angelica sinensis* extract, following ICH Q2 (R1) recommendations. At higher concentrations, no significant differences (*p* > 0.05) were observed between the two methods.

These findings demonstrate that the developed sensing system (AgNCs, PDOA) is more sensitive and has a lower detection limit than the LC-MS method for detecting low concentrations of glyphosate pesticide. Compared to other jobs, the sensors designed and synthesized in this study show better detection limits and recovery rates for glyphosate detection [22]. However, for higher concentrations, both methods provide comparable results. Notably, the LC-MS method requires complex sample preparation, extended analysis time, and expensive specialized equipment [23]. In contrast, sensor technology effectively overcomes these limitations. In conclusion, fluorescent nanosensors show great potential as a rapid and efficient method for detecting glyphosate in real samples of *Angelica sinensis*.

#### 3.3.3. Herbicide Residue Detection of Glyphosate in Field-Cultivated *Angelica sinensis* Samples

**Figure 8:**
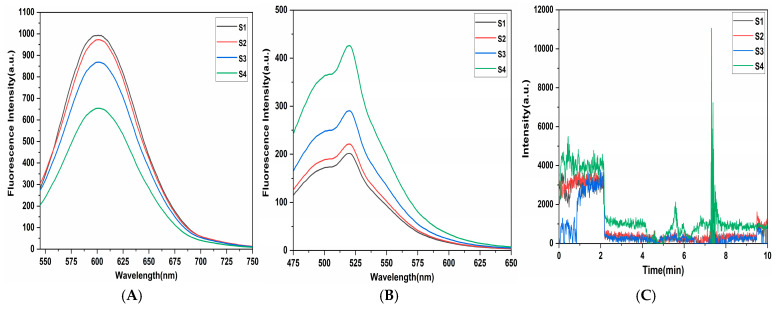
(**A**) Fluorescence spectrum obtained from the PDOA-Cu^2+^ + Glyphosate sensor for detecting glyphosate in field-cultivated *Angelica sinensis*; (**B**) fluorescence spectrum derived from the AgNCs + Glyphosate sensor for glyphosate detection in field-cultivated *Angelica sinensis*; (**C**) total ion chromatogram of glyphosate in field-cultivated *Angelica sinensis* as measured by LC-MS.

To evaluate the practical application of the developed fluorescent nanosensors (AgNCs + Glyphosate, PDOA-Cu^2+^ + Glyphosate), both sensor systems, in conjunction with LC-MS technology, were employed for the rapid and quantitative screening of glyphosate residues in field-cultivated *Angelica sinensis* samples. Figure 8 illustrates the results, while Table 5 presents a comparison of residue/applied percentages.

Glyphosate residues in field-grown *Angelica sinensis* were analyzed using both the sensor technology (AgNCs + Glyphosate, PDOA-Cu^2+^ + Glyphosate) and the LC-MS method. A paired *t*-test was conducted for the results, but the S1 sample could not be analyzed due to incomplete data. For the S2–S4 samples, no significant differences (*p* > 0.05) were observed between the results from the sensor technology and LC-MS detection methods. Specifically, for the S1 sample, glyphosate was detected only by the PDOA-Cu^2+^ + Glyphosate sensor system, with neither the AgNCs sensor system nor LC-MS being able to detect it. This suggests that the PDOA sensor system is more sensitive and has a lower detection limit, making it suitable for detecting ultra-low concentrations of glyphosate residues in *Angelica sinensis*.

For sample S2, both sensor systems and LC-MS detected glyphosate with similar residue/applied values. However, because LC-MS requires longer analysis time and complex sample preparation, it is less suitable for rapid on-site detection. Both sensor systems showed comparable performance for S2, but the PDOA/Cu^2+^ method involved additional steps, making the AgNCs procedure more efficient for this sample.

For samples S3 and S4, glyphosate was successfully detected by both sensor systems and LC-MS, yielding comparable results. The PDOA/Cu^2+^ system is more effective for detecting low concentrations, while the AgNCs system, with its “one-step detection” capability, is better suited for medium concentrations. All three methods are applicable in different scenarios.

As with phoxim residues, the overall residue levels for all methods were relatively low. This may be attributed to possible environmental factors such as rainfall during the growing season or natural metabolic processes of the plant. However, further studies are needed to verify these potential explanations, as no direct environmental or metabolic measurements were performed in this study.

### 3.4. Summary of Analytical Performance of the Three Nanosensors Versus LC-MS

Table 6 compares the calibration parameters, LOD, and LOQ of the three fluorescent nanosensors with those of LC-MS. Although LC-MS exhibited lower instrumental LODs for phoxim (0.0414 μmol·L^−1^) than GQDs@GSH (0.075 μmol·L^−1^), it failed to detect this concentration in real matrix due to severe ion suppression, whereas the nanosensor succeeded. For glyphosate, both PDOA/Cu^2+^ and AgNCs sensors achieved substantially lower LODs (1.8 nmol·L^−1^ and 21 nmol·L^−1^, respectively) than LC-MS (0.146 μmol·L^−1^).

### 3.5. Comparison with Previously Reported Methods

To benchmark the analytical performance of our proposed fluorescent nanobiosensor, we compared its linear range and limit of detection (LOD) with those of previously reported methods for organophosphorus pesticide detection, including surface plasmon resonance (SPR) biosensors, gas chromatography–mass spectrometry (GC-MS), and liquid chromatography–tandem mass spectrometry (LC-MS/MS).

As summarized in Table 7. the GQDs@GSH nanosensor developed in this work achieves a LOD of 26 ng·mL^−1^ for phoxim. This value is significantly lower (i.e., better) than that of SPR biosensors (25 mg·L^−1^ and 2 mg·L^−1^) and is comparable to GC-MS (10 μg·kg^−1^) and LC-MS/MS (0.005 mg·kg^−1^). Moreover, our method offers advantages in terms of simplicity, rapid detection (~10–15 min), and cost-effectiveness compared to instrumental methods such as GC-MS and LC-MS/MS.

These results demonstrate that the proposed fluorescent nanosensor is a competitive alternative for rapid and sensitive detection of organophosphorus pesticide residues in Angelica sinensis.

## 4. Conclusions

This study investigated the impact of nanomaterial intake on the growth of *Angelica sinensis*, focusing on the detection of organophosphorus pesticide residues using both fluorescent nanosensors and liquid chromatography–mass spectrometry (LC-MS). The results of pesticide residue tests for phoxim and glyphosate, conducted in both spiked and field conditions, demonstrated that the fluorescent nanosensor systems (GQDs@GSH, AgNCs, PDOA) offer a simpler, more sensitive alternative to LC-MS for detecting low concentrations of pesticide residues in *Angelica sinensis*.

For phoxim, both methods provided comparable recovery rates at medium and high concentrations. However, the fluorescent sensor method exhibited superior sensitivity and a lower detection limit for low concentrations, making it ideal for field use. The GQDs@GSH system, in particular, proved effective at detecting lower pesticide residues, offering advantages in cost and simplicity over LC-MS, which requires expensive equipment and complex sample preparation.

Similarly, for glyphosate detection, the fluorescence sensors (AgNCs + Glyphosate, PDOA-Cu^2+^ + Glyphosate) demonstrated comparable or superior performance in detecting low concentrations compared to LC-MS. The PDOA-Cu^2+^ + Glyphosate sensor, with its enhanced sensitivity, was the only system able to detect glyphosate in the S1 sample. While both sensor systems and LC-MS provided comparable results for higher concentrations (S2 sample), the sensor systems were more suitable for rapid on-site detection, while LC-MS was hindered by longer detection times and more complex procedures.

A seemingly contradictory observation—low LC-MS recoveries (11–25%) alongside excellent nanosensor performance—actually reveals a fundamental insight that extends beyond pesticide analysis. LC-MS remains the gold standard, but its accuracy presupposes optimal sample purification and matrix-matched calibration. In complex matrices such as *Angelica sinensis* root, co-extracted interferents cause severe ion suppression (ME = −72%), compromising recovery even under pharmacopeia-compliant protocols. Nanosensors, relying on fluorescence rather than ionization, bypass this limitation entirely. Thus, the low LC-MS recoveries do not invalidate LC-MS; rather, they delineate its boundary conditions and, conversely, define the unique value proposition of nanosensors: matrix-robust, rapid, and sensitive screening at the cost of structural information. This complementary relationship—not competition—forms the core take-home message, positioning nanosensors as orthogonal tools rather than substitutes for LC-MS. For routine surveillance of low-level pesticide residues in herbal medicines, nanosensors offer a fit-for-purpose alternative; for confirmation and unknown identification, LC-MS remains irreplaceable. The originality of this work is threefold: it is the first application of GQDs@GSH, PDOA, and AgNCs for pesticide detection in Angelica sinensis; the first systematic comparison with pharmacopeia-standard LC-MS in this complex matrix; and the first phytotoxicity assessment of these nanosensors in planta. This paradigm extends beyond pesticide analysis to any analytical challenge where matrix complexity challenges traditional platforms, offering a generalizable framework for orthogonal method validation.

## Figures and Tables

**Table 1:** Optimized MRM parameters for phoxim and glyphosate detection by LC-MS.

Analyte	Retention Time (min)	Precursor Ion (*m*/*z*)	Production (*m*/*z*)	Collision Energy (eV)
Phoxim	7.13	299.1	129.0 (quant), 77.0 (qual)	22, 35
Glyphosate	5.56	170	88.0 (quant), 60.0 (qual)	18, 30

**Table 2:** Comparison of GQDs@GSH system and LC-MS method for the determination of phoxim pesticide residues in *Angelicae sinensis*.

Method	Addition Level (μmol·L^−1^)	Measured Value (n = 3)	Recovery Rate (%)	RSD (%)
GQDs@GSH	Low (0.0414)	0.0404 ± 0.0014	97.65	3.35
	Medium (0.21)	0.2104 ± 0.0043	100.18	2.06
	Medium (0.4138)	0.4126 ± 0.0083	100.62	2.00
	High (0.83)	0.8311 ± 0.0041	100.13	0.49
LC-MS	Low (0.0114)	—	—	—
	Medium (0.21)	0.0234 ± 0.00035	11.12	3.78
	Medium (0.4138)	0.0643 ± 0.0010	15.54	1.28
	High (0.83)	0.2138 ± 0.0022	25.46	0.25

Note: The GQDs@GSH system demonstrated higher recovery rates and lower RSD values compared to the LC-MS method, indicating superior sensitivity and precision, particularly for low-concentration phoxim residues.

**Table 3:** Comparison of phoxim detection results in field-grown *Angelica sinensis* samples using the GQDs@GSH sensor system and LC-MS method.

Method	Addition Level (μmol·L^−1^)	Measured Value (n = 3)	Residue/Applied (%)
GQDs@GSH	S1 Low (32)	0.0384 ± 0.000265	0.12
	S2 Medium (64)	0.0422 ± 0.0036	0.66
	S3 Medium (96)	1.3362 ± 0.0047	1.39
	S4 High (128)	0.8311 ± 0.0041	1.08
LC-MS	S1 Low (32)	_	_
	S2 Medium (64)	0.422 ± 0.00673	0.66
	S3 Medium (96)	1.3362 ± 0.00878	1.39
	S4 High (128)	1.383 ± 0.01426	1.08

Note: Recovery rate (%) = (measured residue concentration in root/applied pesticide dose) × 100%. This reflects detectable residue level relative to total applied dose, not analytical spike recovery.

**Table 4:** Comparison of the results of the detection of glyphosate residues in *Angelicae sinensis* by AgNCs, PDOA sensing system, and LC-MS method.

Method	Addition Level (μmol·L^−1^)	Measured Value (n = 3)	Recovery Rate (%)	RSD (%)
PDOA Method	Low (0.146)	0.1449 ± 0.0021	99.27	2.341
	Medium (0.219)	0.23312 ± 0.00945	105.34	0.849
	Medium (0.292)	0.3078 ± 0.0031	100.04	1.09
	High (0.365)	0.3581 ± 0.0026	102.95	1.605
AgNCs Method	Low (0.146)	0.1231 ± 0.0047	91.3	3.345
	Medium (0.219)	0.2218 ± 0.00673	101.29	2.106
	Medium (0.292)	0.1927 ± 0.00454	100.23	1.556
	High (0.365)	0.3640 ± 0.0044	99.71	1.219
LC-MS methods	Low (0.146)	0.1228 ± 0.00153	84.07	1.245
	Medium (0.219)	0.2355 ± 0.00578	106.78	2.463
	Medium (0.292)	0.3028 ± 0.01008	105.42	3.272
	High (0.365)	0.3608 ± 0.01162	98.12	3.247

**Table 5:** Comparison of glyphosate determination results in field-cultivated *Angelica sinensis* by AgNCs + Glyphosate, PDOA-Cu^2+^ +Glyphosate, and LC-MS methods (μmol·L^−1^).

Method	Addition Level (μmol·L^−1^)	Measured Value (n = 3)	Residue/Applied (%)
PDOA Method	Low (61.74)	0.0036 ± 0.00006	99.27
	Medium (123.48)	0.094 ± 0.001	0.0058
	Medium (185.22)	0.419 ± 0.003	0.076
	High (246.96)	1.057 ± 0.0042	0.428
AgNCs Method	Low (61.74)	—	—
	Medium (123.48)	0.094 ± 0.003	0.076
	Medium (185.22)	0.419 ± 0.005	0.226
	High (246.96)	1.059 ± 0.0042	0.429
LC-MS methods	Low (61.74)	—	—
	Medium (123.48)	0.094 ± 0.002	0.002
	Medium (185.22)	0.419 ± 0.00557	0.226
	High (246.96)	1.058 ± 0.01411	0.428

Note: Residue/applied (%) = (measured concentration in root/applied pesticide dose) × 100%. This metric reflects the detectable residue level relative to the total amount applied, not analytical spike recovery.

**Table 6:** Summary of key validation parameters for nanosensors and LC-MS.

Parameter	GQDs@GSH (Phoxim)	PDOA/Cu^2+^ (Glyphosate)	AgNCs (Glyphosate)	LC-MS (Phoxim)	LC-MS (Glyphosate)
Calibration range (μmol·L^−1^)	0.1–10.0	0–1.5	0–3.0	0.1–10.0	0.1–5.0
Regression equation	y = 0.2005x + 0.0572	y = 1.1095x + 0.6665	y = −0.3244x + 1.0094	y = 679,642 + 102,896x	y = 32,596x − 56,102
R^2^	0.9995	0.9973	0.9972	>0.99	>0.99
LOD (μmol·L^−1^)	0.075	0.0018	0.021	0.0414	0.146
LOQ (μmol·L^−1^)	0.0414 *	0.146 *	0.146 *	0.0414	0.146

* LOQ experimentally validated at the lowest spiked concentration showing acceptable recovery and RSD.

**Table 7:** Comparison of analytical performance of proposed nanosensors with previously reported methods for organophosphorus pesticide detection.

Method	Analyte	LOD	Linear Range	Response Time	Reference
SPR biosensor	Phoxim	2 mg·L−1	0–1 mg·L−1	–	[11]
GC-MS	Phoxim	10 μg·kg−1	5–5000 μg·kg−1	–	[12]
GQDs@GSH	Phoxim	0.075 μmol·L−1	0.1–10.0 μmol·L−1	80 min	This work
PDOA/Cu2+	Glyphosate	1.8 nmol·L−1	0–1.5 μmol·L−1	30 min	This work
AgNCs	Glyphosate	21 nmol·L−1	0–3.0 μmol·L−1	1 min	This work

## Data Availability

Experimental data associated with this research are available from the authors.

## Supplementary Materials

The following supporting information can be downloaded at: https://www.mdpi.com/article/10.3390/bios16060311/s1.
