# Supplementary material for: Novel Fluorescent Nanobiosensors for Rapid and Sensitive Detection of Organophosphorus Pesticide Residues in Angelica sinensis: A Performance Evaluation Against LC-MS
Source: Biosensors (Basel). 2026 Jun 1;16(6):311. doi: 10.3390/bios16060311 (PMC13297375; doi:10.3390/bios16060311)
Supplement: Supplementary file 1 [file biosensors-16-00311-s001.zip › biosensors-4281477-Supplementary.pdf]

biosensors

MDPI

Supplementary materials

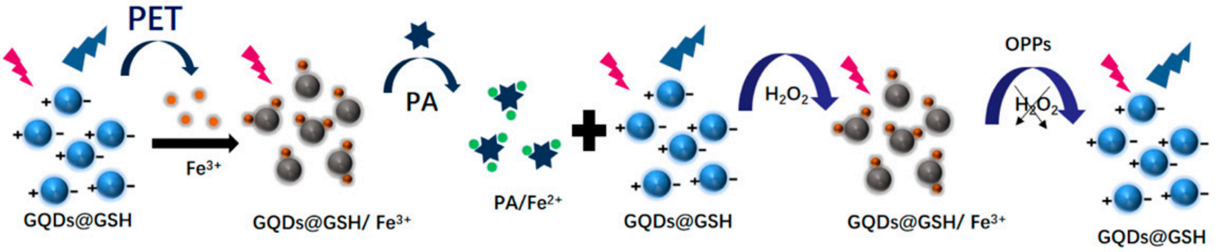

Figure S1. A. Schematic "off-on-off" fluorescence switching mechanism of the GQDs@GSH/Fe^{3+}/PA/(AChE/ChOx) system for OPP detection. Fe^{3+} induces PET quenching (off), PA restores fluorescence (on), and H_2O_2 (from ACh hydrolysis) re-quenches (off). OPPs inhibit AChE, block H_2O_2 generation, and maintain the "on" state.

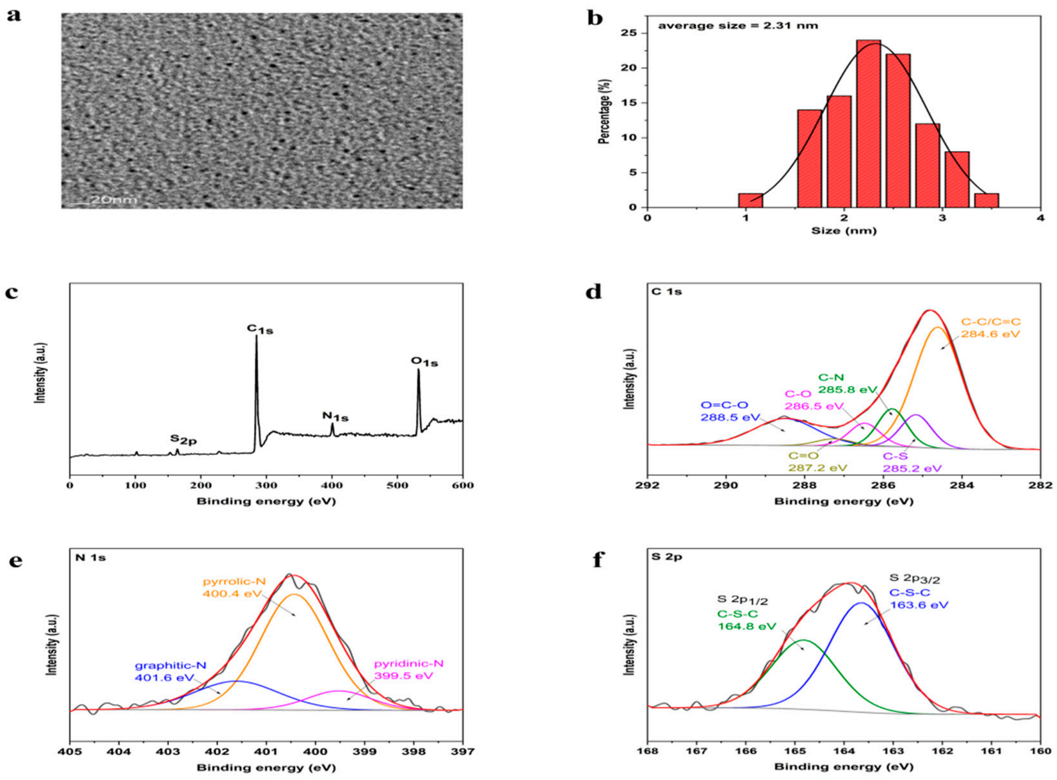

Figure S2. Morphology, size distribution and elemental chemical state characterization of the GQDs@GSH.(**a**) Transmission electron microscope (TEM) image of the as-synthesized nanoprobe.(**b**) Statistical histogram of nanoparticle average size distribution.(**c**) Total survey X-ray photoelectron spectroscopy (XPS) spectrum.(**d**) High-resolution XPS spectrum of C 1s orbital.(**e**) High-resolution XPS spectrum of N 1s orbital.(**f**) High-resolution XPS spectrum of S 2p orbital.

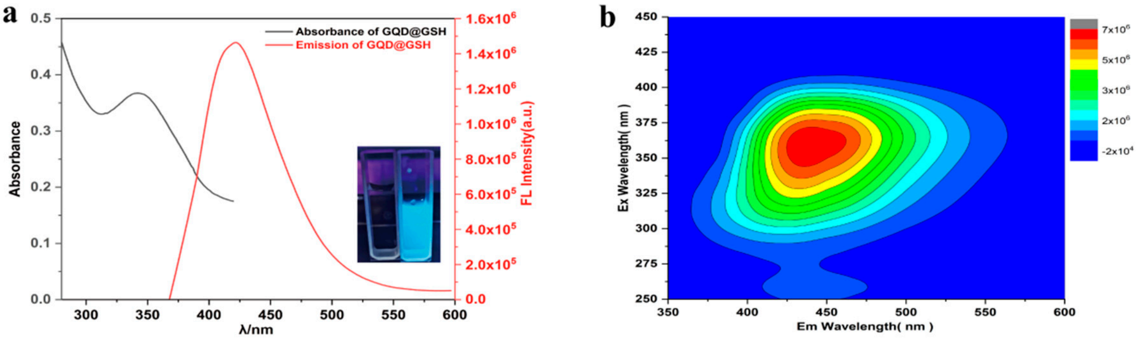

Figure S3. Optical characterization of GQDs@GSH.(**a**) UV-vis absorption and fluorescence emission spectra (inset: photographs under daylight and UV light).(**b**) Fluorescence 3D contour map (excitation-emission matrix).

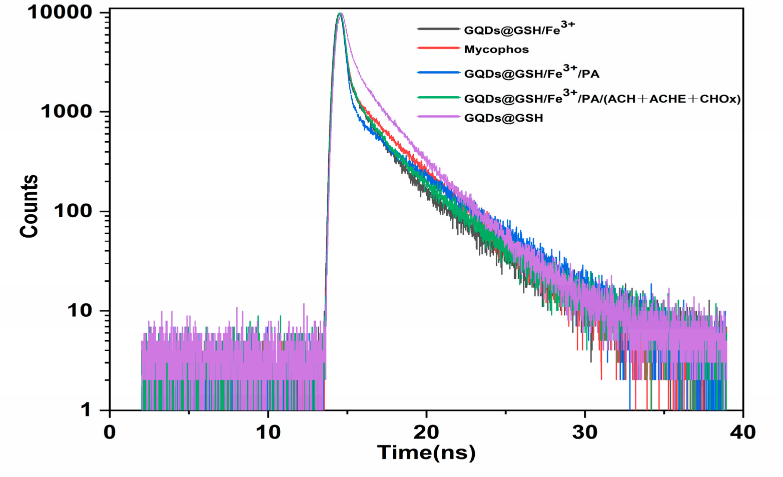

Figure S4. Fluorescence decay curves of GQDs@GSH, GQDs@GSH/Fe^{3+}, GQDs@GSH/Fe^{3+}/PA, and GQDs@GSH/Fe^{3+}/PA/(ACh + AChE + ChOx). The shortest lifetime was observed for Fe^{3+}-quenched samples, while PA treatment recovered the decay time.

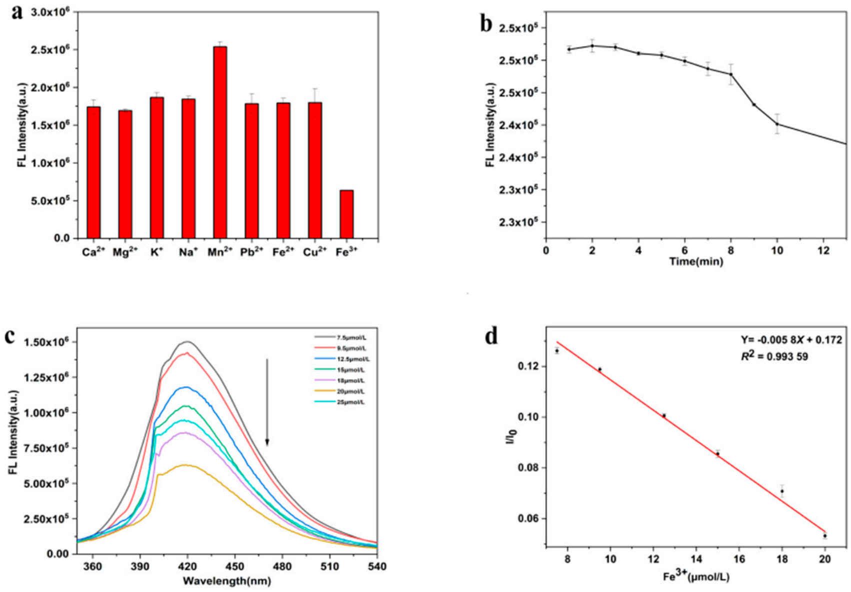

Figure S5. Full-range emission spectrum of PDOA/Cu^{2+} probe.

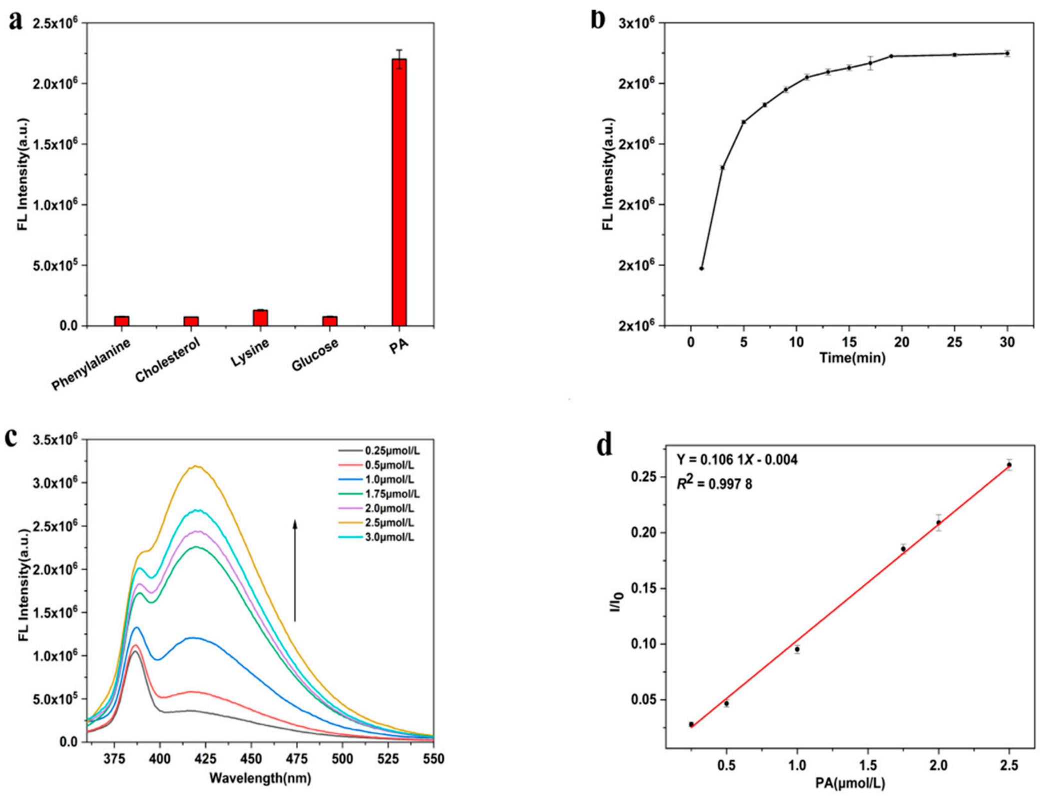

Figure S6. Fluorescence characterization of the probe.(**a**) pH stability of the probe.(**b**) Time-dependent fluorescence response.(**c**) Fluorescence emission spectra with increasing pesticide concentration.(**d**) Linear calibration curve.

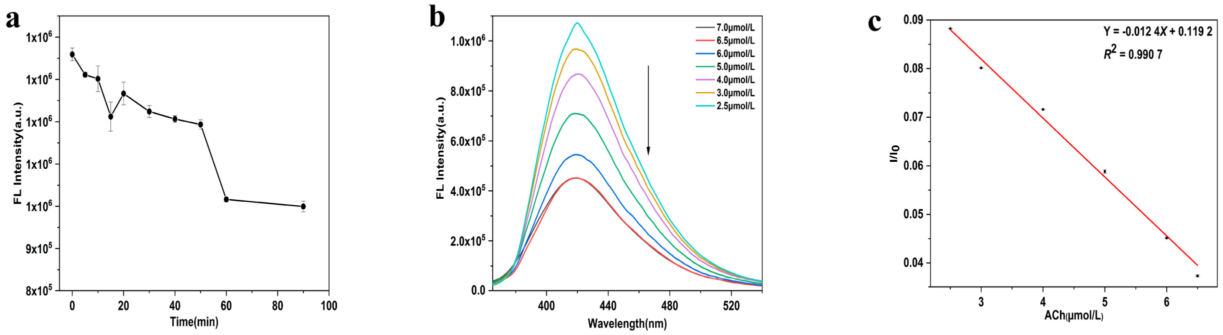

Figure S7. Fluorescence intensity changes (I/I_0) over time (or wavelength) for three different conditions (**a, b, c**). The data demonstrate the quenching and recovery behavior of the sensor in response to glyphosate and interfering substances.

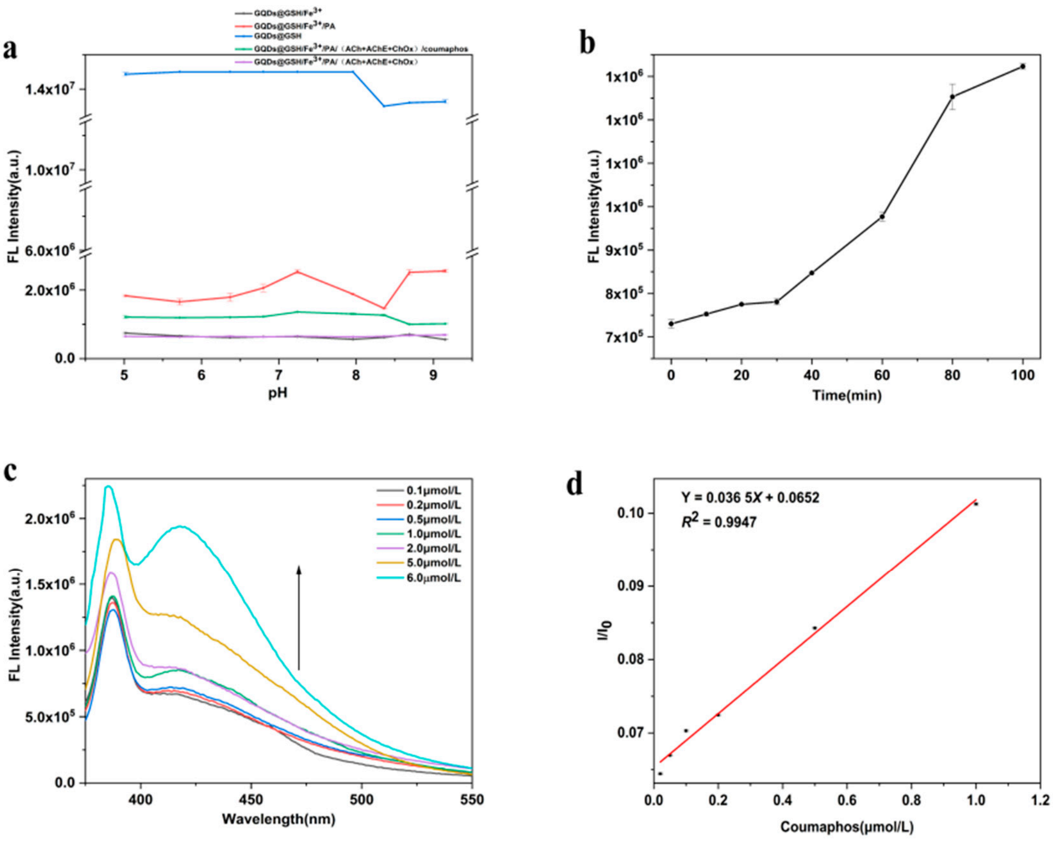

Figure S8. Fluorescence characterization of the probe.(**a**) pH stability of the probe.(**b**) Time-dependent fluorescence response.(**c**) Fluorescence emission spectra with increasing pesticide concentration.(**d**) Linear calibration curve.
